# Optimisation of Interfacial Modification by Two-Stage Microwave Irradiation

**DOI:** 10.3390/molecules29153673

**Published:** 2024-08-02

**Authors:** Yusuke Asakuma, Yusuke Watanabe, Anita Hyde, Chi Phan

**Affiliations:** 1 Department of Chemical, Energy and Environmental Engineering, Kansai University, 3-3-35 Yamate-cho, Suita 564-8680, Japan; 2 Department of Chemical Engineering, University of Hyogo, Shosha 2167, Himeji 671-2280, Japan; yusuke_asakuma@yahoo.co.jp; 3 Department of Chemical Engineering, Curtin University, Perth, WA 6845, Australia; anita.hyde@gmail.com

**Keywords:** microwave, de-emulsification, desorption, non-ionic surfactant

## Abstract

Microwave-assisted de-emulsification is attractive in the processes of petroleum production and refining. The main advantage of microwaves is their direct influence on the surfactant layer at the oil/water interface. Previously, an effective interfacial modification was demonstrated by pulsed microwave irradiation. However, the effect of the modification diminished during the off interval of the pulse irradiation. In this study, two-stage microwave irradiation with different powers and durations was applied as a method to maintain an interfacial effect. The power of the second stage was changed to optimise the modification. Quick modification was obtained by high-power irradiation followed by low-power irradiation. It was confirmed a sustained modification was maintained by a moderate power of the second irradiation. This observation indicates a re-adsorption or re-structure process after the first irradiation is suppressed by the second irradiation. The results open new opportunities to optimise microwave operation in oil/water systems.

## 1. Introduction

In multiple-phase fluids, microwaves can pass through the non-polar phase and directly reach the polar liquid. Consequently, a polar molecule such as water absorbs the microwave directly, and the molecules near the interface are vibrated or rotated significantly. As a result, microwave heating is faster and more energy-efficient than conventional heating. Furthermore, at an oil/water interface, microwaves can have additional heating effects due to the differences in thermal and dielectric properties between these components. The effect is particularly strong for a surfactant layer at the oil/water interface. Such a layer is very important to stabilise or destabilise emulsions.

The use of microwaves has been proposed as part of a new de-emulsification technique for petroleum production and refining due to the thermal energy concentration at the oil/water interface [1,2,3]. In contrast, conventional heating requires significant energy to heat the oil phase, which remains largely unheated in microwave operation. Energy efficiency is particularly high for mixtures with a high oil content, such as crude oil with small dispersed water droplets [4]. Microwave-assisted interfacial modifications are also beneficial to oil extraction from natural products [5,6]. The food industries also employ non-ionic surfactants to generate food-grade emulsions [7]. In this case, undesirable de-emulsification may reduce food qualities such as taste and aroma. Similarly, microwave-assisted extraction can improve the efficiency of bioactive component production in the pharmaceutical industry [8]. Microwaves can also enhance solid/oil separation and reduce agricultural waste [9].

In the above processes, modification at the interface results in the quick thermal response of microwave absorbance [10,11,12,13,14], which is not seen with slower thermal conduction. Using these characteristics, undesirable emulsions formed during petroleum production and refining can be removed efficiently. In these instances, microwave heating can cause the surfactant layer to desorb from the surface and thus reduce surface viscosity and enhance phase separation [8].

**Figure 1:**
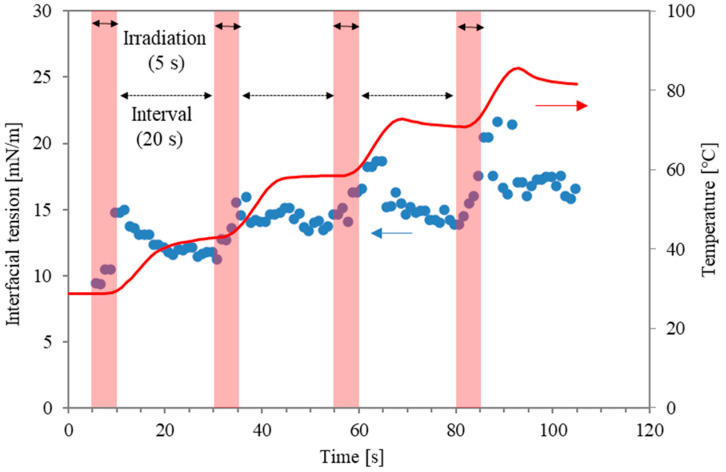
Interfacial tension profiles and temperature as a function of time. The red arrows refer to temperature on the right axis, and the blue arrows refer to interfacial tension on the left axis.

On the other hand, the prevention of unstable operations, such as the boiling behaviour caused by microwave local heating, remains a challenge. Recently, a dimensionless number related to the energy concentration of microwave absorbance was proposed to characterise stable operation, prevent boiling behaviour, and clarify the mechanism of the interfacial modification [14,15]. In the case of continuous microwave irradiation, the overheating effect can make the interfacial modification less effective than conventional heating. For example, local boiling with micro-bubbles in the water phase can occur earlier than surfactant desorption. Accordingly, to optimise the special effects of microwaves for surfactant desorption around the interface, pulsed irradiation has been proposed in previous studies [12,13,14]. Figure 1 shows an example of interfacial tension between water with a surfactant (Triton X-100; 0.2 mM, Kishida Chemical Co., Osaka, Japan) and n-decane during pulse irradiation when the irradiation time and non-irradiation time were 5 s and 20 s [16]. The data represent temperature and interfacial tension, respectively. Although quick modification was obtained during the irradiation time (as shown in the red areas), it was inevitable that the interfacial tension was reduced during the non-radiation intervals. In other words, surfactant molecules from the interface that are desorbed or disordered due to the microwaves will be re-adsorbed or re-structured during the non-irradiation intervals. Accordingly, the irradiation pattern can be improved to prolong the interfacial modification level. It is noteworthy that the microwave power in the previous study was maintained at the same level for all irradiation periods.

In addition to exposure time, another advantage of microwave reactors is the ability to fine-tune power. The the flexibility of microwave sources enables tailored heating profiles for specific materials and reactions. In this study, two-stage irradiation, which consists of a higher power and subsequent lower power, was employed to examine this effect. The two-staged heating with different power levels offers flexibility to optimize the operation. In the on–off pulse irradiation mentioned above, the microwave effect disappears during the intervals. The proposed two-stage irradiation intends to compensate for this drawback while avoiding undesirable boiling. Ultimately, the study aims to optimize heating operations—that is, maximize the interfacial modification while limiting local boiling.

## 2. Results

The experiment consists of three sets of conditions. The first set includes a constant first irradiation period of 5 s and 480 W, followed by a second stage with a different level of power. The second set consists of continuous irradiation. The third set consists of a variable first stage of irradiation followed by a constant second stage. The details of power levels and irradiation times are listed in Table 1, Table 2 and Table 3, respectively. The total energy in all experiments was maintained at 4800 J.

**Figure 2:**
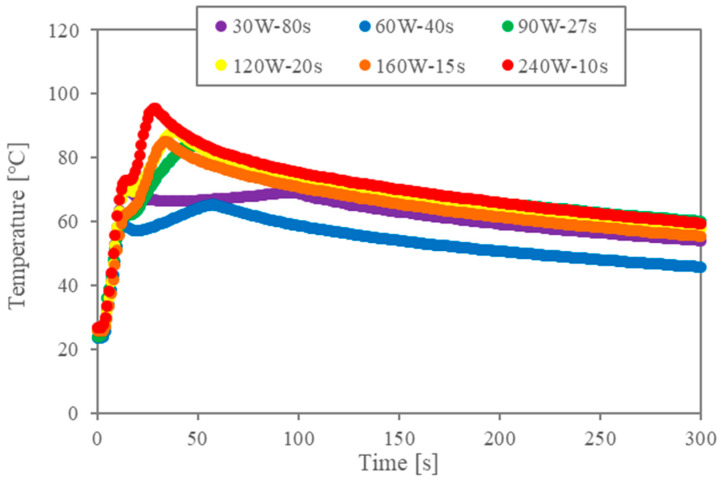
Temperature profiles for two-stage irradiation trials (in all instances, the first stage consisted of 5 s of irradiation at 480 W).

First, the temperature data are analysed to validate the impact of the multiple-stage microwaves. Figure 2a shows the temperature profiles for the two-stage irradiation (corresponding to Table 1). The legend indicates the conditions for the second stage of irradiation when the first irradiation is 480 W and 5 s. Since the power of the first stage is large, the temperature rises rapidly under all conditions. Microwaves are absorbed by the cell walls and the liquid–liquid interface, and the temperature continues to rise during the second irradiation due to both the thermal conduction of heat generation by the first generation and the heat generation of the second irradiation. However, when the power of the second irradiation is small, the higher temperature obtained by the first irradiation cannot be maintained. In any case, the temperature reaches maximum temperature and decreases slowly after the second irradiation is turned off. Although the total energy is the same, the maximum temperature and the time to reach it are different.

In previous studies [10,11,12,13,14,16], levels of interfacial tension during microwave heating and conventional heating were compared through the relation between interfacial tension and temperature. The energy concentration of the microwaves during the irradiation at the interface is essential for the interfacial modification. Accordingly, the temperature profiles in Figure 2 show that the energy concentration becomes lower when the second power level is lower.

**Figure 3:**
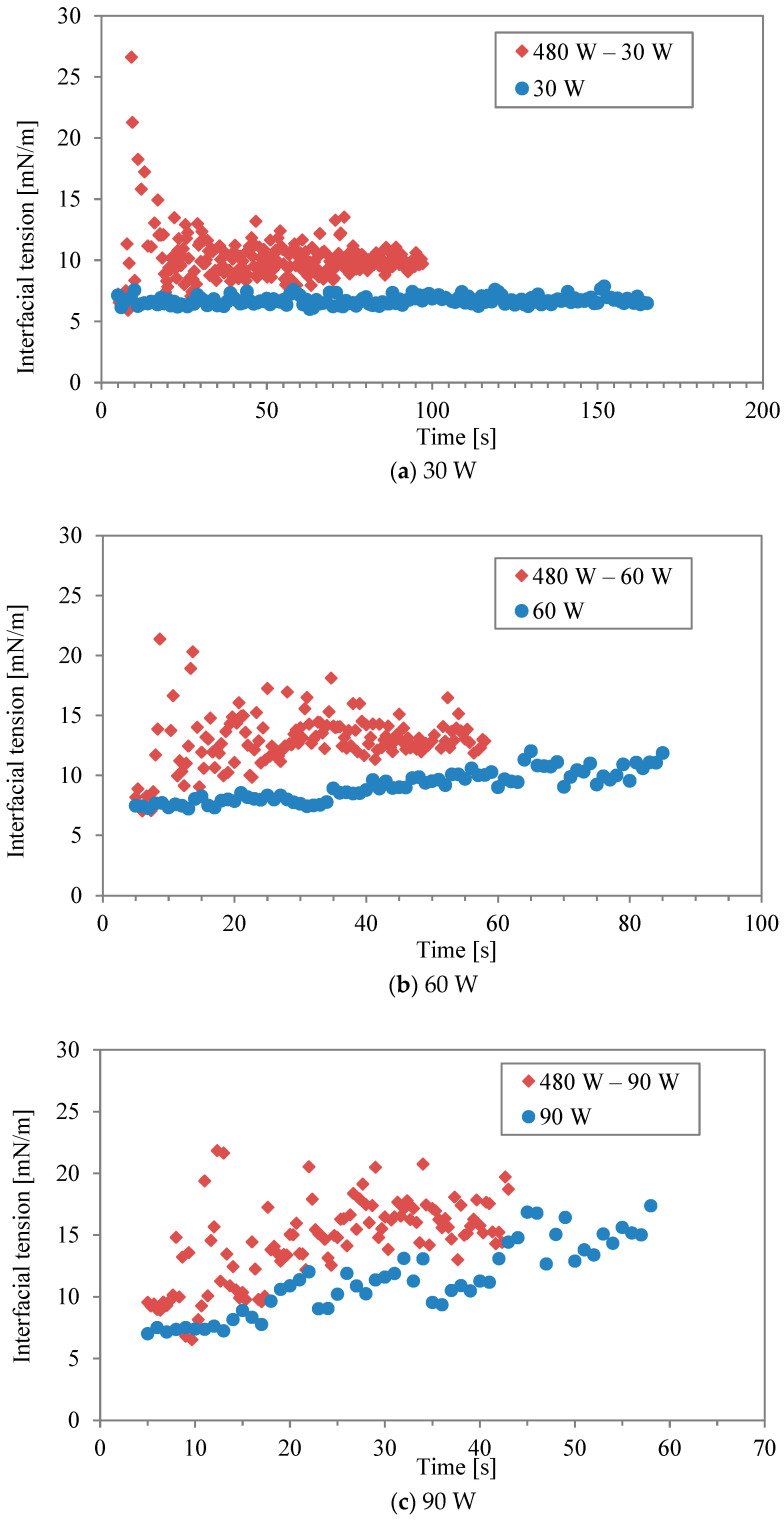

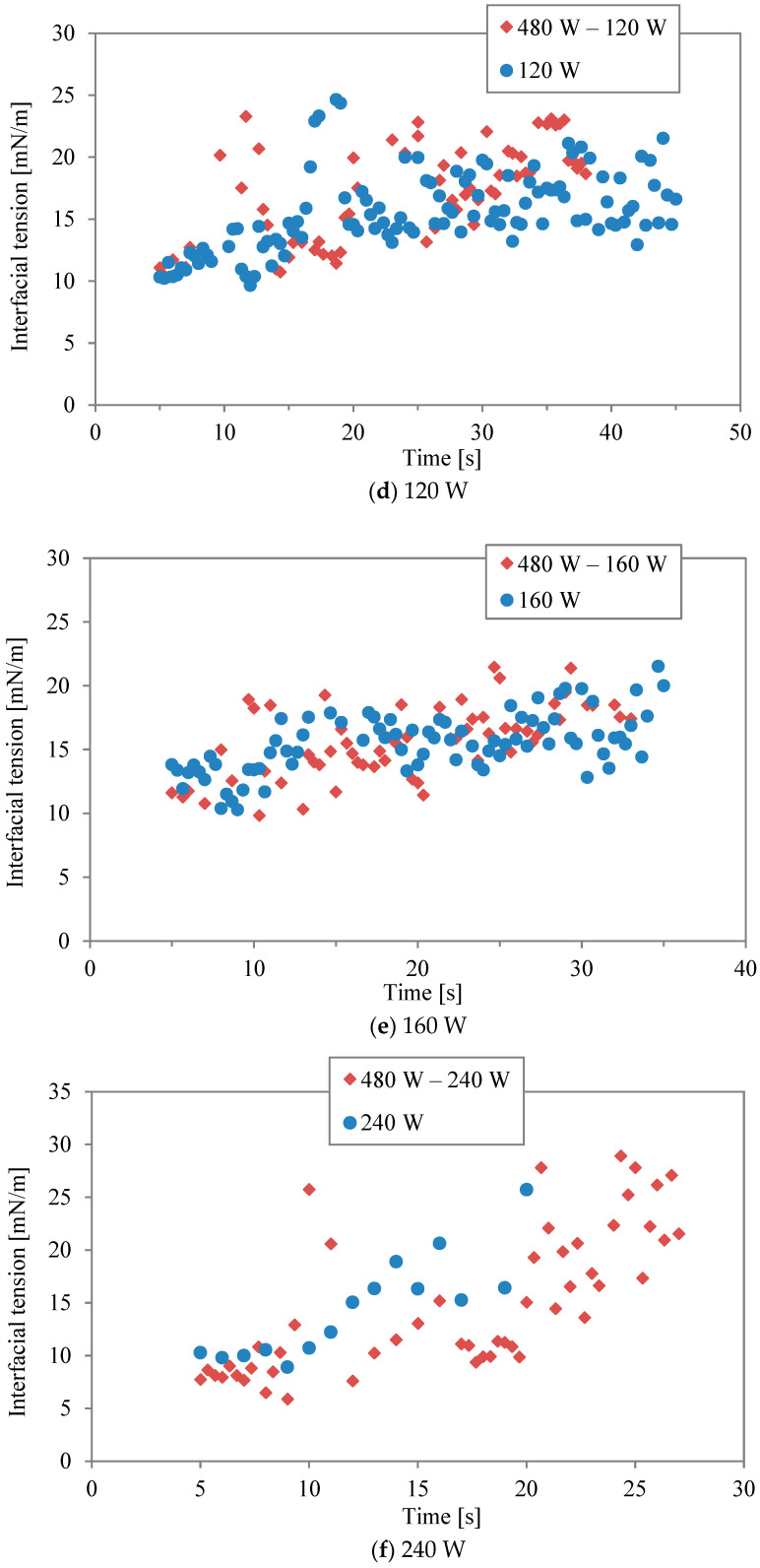
Interfacial tension between two-stage irradiation and continuous irradiation at different power levels: (**a**) 30 W, (**b**) 60 W, (**c**) 90 W, (**d**) 120 W, (**e**) 160 W, and (**f**) 240 W.

Figure 3a–f show comparisons of interfacial tension between two-stage irradiation and continuous irradiation for the different power levels listed in Table 1 and Table 2. Generally, the water/decane tension increases with rising temperature, as observed with conventional heating [17]. Regarding microwave heating, it has been argued that the interaction between water and the hydrophilic part, comprising ethylene oxide units of Triton X-100, is strongly disrupted by microwaves [16], leading to surface desorption. It should be noted that the accurate measurement of interfacial tension at 240 W (in continuous mode) was impossible during the last 5 s of the irradiation due to boiling. At lower powers of 30 W and 60 W (Figure 3a,b), the interfacial tension in the two-stage irradiation is higher than that in the continuous mode, although the total energy is the same. It was found that the effect of the interfacial modification obtained by the first higher power during the two-stage irradiation could be maintained even at the lower power. In other words, while higher irradiation power had a more significant effect on interfacial tension, a second irradiation period at lower power can be sufficient to interrupt the re-structure and re-adsorption of surfactant molecules, maintaining the effect achieved by the initial irradiation. This is most clearly seen in Figure 3f, where continuous irradiation at 30 W induces little response in the interfacial tension. However, when following an initial irradiation of 480 W, 30 W is sufficient to maintain a level of change in the interfacial tension. On the other hand, the effect becomes minimal as “low” power is increased. For example, the power in continuous mode (120 W and 240 W) was sufficiently high for surfactant desorption in the interfacial modification.

**Figure 4:**
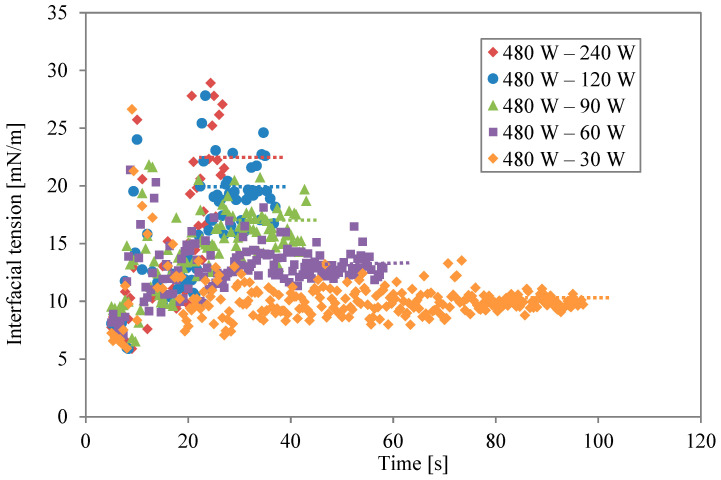
Interfacial tension profiles for different power levels during second irradiation.

Data on the two-stage irradiation at different power levels are plotted simultaneously in Figure 4. The broken lines are the average values for the last 5 s of the second irradiation. Changing the level of microwave power and irradiation time altered the concentration of thermal energy at the solution interfaces. The level of interfacial modification is different, even if the same energy is irradiated. When the second irradiation power was lower, the modification was weak. However, the effect of the modification lasted for a long time when the same energy was irradiated. As the power became higher, in contrast, the effect was stronger and shorter.

**Figure 5:**
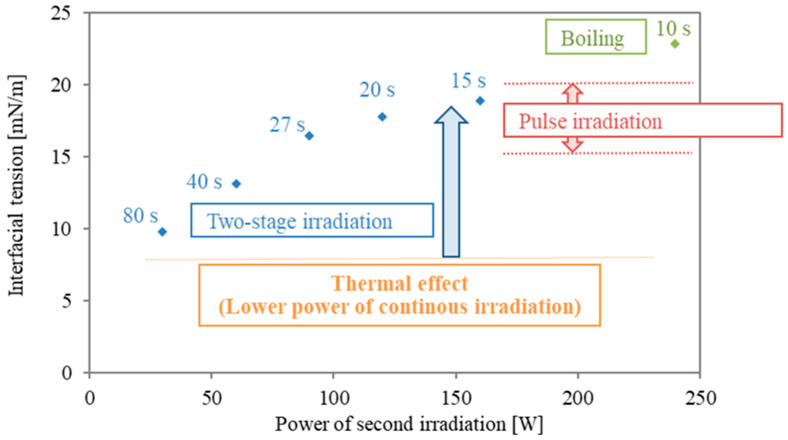
Summary of interfacial modification with different irradiation modes in the second irradiation. The final interfacial tension of the various two-stage modes from Table 1 No. 1–5 are presented in blue, in comparison with the range of interfacial tension results from pulsed irradiation (Figure 1) in red. The last of the two-stage experiments, Table 1 No. 6, evidenced boiling and is shown in green.

Figure 5 presents a summary of the interfacial modification at different irradiation modes. The values in Figure 5 are averaged interfacial tension rates of the last 5 s of irradiation time and are plotted for the second power stage of the two-stage irradiation. The value of 240 W is shown for reference, although boiling happened boiling occurred at the interface. As shown in Figure 1, in the case with a higher power of pulse irradiation, an increment of 15–20 mN/m was obtained. However, the duration is less than 5 s, and the period of interfacial modification is short. As shown in Figure 3a, continuous irradiation at lower power (30 W) was almost similar to the data from conventional heating, and interfacial tension remained mostly constant. This means that surfactant desorption is quite difficult with only thermal effects (heating). As the power in the second stage of the two stages is higher, the interfacial tension during the second irradiation becomes larger. The energy concentration of microwaves is essential for interfacial modification, and the following second irradiation plays an important role in the prevention of the re-adsorption and re-structure of surfactant molecules.

**Figure 6:**
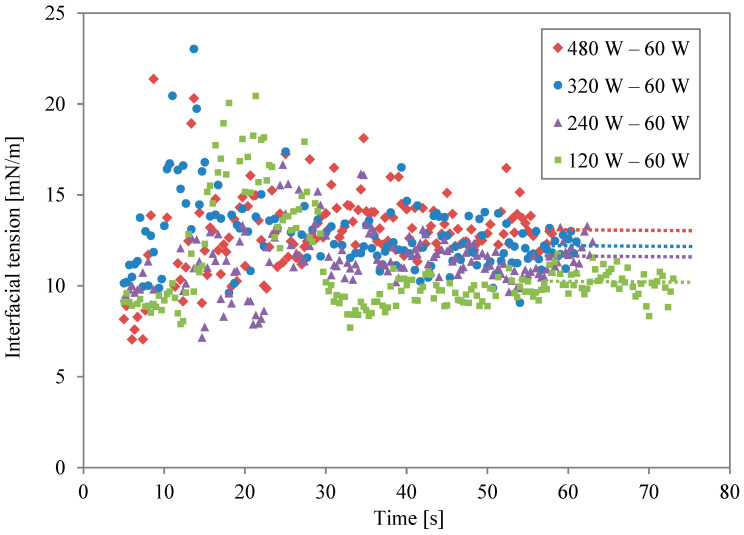
Interfacial tension profiles for different power levels in the first irradiation.

Figure 6 shows the interfacial tension for different irradiation levels in the first power stage, following the conditions listed in Table 3. The dot lines are averaged interfacial tension rates from the last 5 s. The power of the first irradiation in two-stage irradiation is an important factor for final interfacial tension as well. However, comparing Figure 4 and Figure 6, the power of the second stage is more important for obtaining stable and higher interfacial tension. Prolonged periods of moderate surface enhancement might be beneficial to oil/water systems with natural surfactants. For example, it has been shown that the adsorption of surfactant at the oil/water interface may take up to 1 h to reach equilibrium [4]. Furthermore, for a practical de-emulsification process, the reduced surface shear viscosity needs to be maintained for a sufficiently long period [18]. Crude oil emulsions are also stabilized by high-molecular-weight compounds known as asphaltenes [19], which take a long time to unfold and desorb from the surface. Another advantage of slow heating is the required time to heat the oil phase between two water-in-oil emulsions. In practice, de-emulsifiers are employed to displace natural asphaltenes. The efficiency of a de-emulsifier depends on molecular structure, as well as temperature. For instance, it has been found that the efficiency of non-ionic de-emulsifiers increases with increasing temperature up to 70 °C [20]. Slow and multiple-staged heating can also help to maintain temperature at an optimal value [4,21]. It has been found that the local movement of the surfactant layer can significantly displace the oil/water interface and affect droplet behaviour [22]. In summary, combinations of repeated cycles, intensity, and microwave power allow flexibility to optimise heating and surface modification times for different applications [23].

## 3. Materials and Methods

N-decane (99%) and Triton X-100 were supplied by Kanto Chemical Co., Tokyo, Japan and Kishida Chemical Chemical Co., Osaka, Japan, respectively. The procedures of experimental setup for interfacial tension measurement and apparatus setup are described in a previous study [10]. Surfactant aqueous solution (heavy phase) and n-decane (light phase) were poured into a square quartz cell with sides measuring 3 cm × 3 cm × 3 cm. The volumes of the water and oil phases were 5 mL and 2 mL, respectively. The cell was placed in the centre of the microwave reactor. A Teflon ball of 6.35 mm diameter was immersed to deform the meniscus for the optical measurement of interfacial tension [15]. The ball was affixed by a Teflon tube (outer diameter of 2 mm), and the position was adjusted to make the interface symmetric. An optical fibre thermometer penetrated through the tube and ball. The temperature around the fibre’s tip, which was set on the bottom of the ball in the aqueous phase, was measured during and after microwave irradiation. The shape was captured by a camera, which was located at the side of the reactor. Triton X-100 was applied in an amount of 0.2 mM. The concentration was above its respective critical aggregate concentration (CAC) [24].

Two-stage irradiation was conducted with the aim of maintaining the effect of the interfacial modification obtained by the first irradiation of higher power while preventing boiling. First, irradiation with a high power (480 W) and short irradiation time (5 s) was carried out. Secondly, microwaves with a lower power and longer irradiation time were used. The conditions used in the experiments are listed in Table 1. The power and irradiation time of the second irradiation were decided so that the total microwave energy remained the same (4800 J) for all conditions. The effect of the second irradiation on interfacial tension was investigated. Moreover, continuous irradiation was performed with the same power as the second irradiation of the two-stage mode, as listed in Table 2. Finally, the power of the first irradiation was changed at the same total energy (4800 J) when the second power was fixed at 60 W, as listed in Table 3.

## 4. Conclusions

Two-stage irradiation was carried out to maintain the effect of interfacial modification by microwave irradiation. First, a high power was irradiated for a short time, and a quick response of interfacial tension was confirmed. After that, second irradiations of various powers were conducted. When the second power was lower, the effect of the first irradiation could be maintained to some extent. Even when the second irradiation power was lower, re-ordering of the disordered or desorbed surfactant molecules established by the first irradiation could be delayed, maintaining changes to the interfacial tension. Although the second power was smaller, interfacial modification could be maintained for a long time. On the other hand, when the second-stage power was higher, a stronger modification effect was observed. The modification level was notably higher than that seen with the thermal effect, and the special microwave effect was confirmed. However, the effect did not last for a long time, and finally, boiling occurred. Accordingly, when the second power was at a medium level (around 60 W and 90 W), the relatively stronger effect lasted for about 1 min. Moreover, it was found that the second power is more important than the first power to modify interfacial tension. This study shows that two-stage irradiation with different power rates is more effective than single-stage or cyclic stages with the same microwave power. The results open new opportunities to optimise microwave operation in oil/water systems.

## Figures and Tables

**Table 1:** Experimental conditions for different levels of secondary power in two-stage irradiation.

No.	Power of First Irradiation [W]	Irradiation Time of First Irradiation [s]	Power of Second Irradiation [W]	Irradiation Time of Second Irradiation [s]	Energy [J]
1-1	480	5	30	80	4800
1-2	480	5	60	40	4800
1-3	480	5	90	27	4800
1-4	480	5	120	20	4800
1-5	480	5	160	15	4800
1-6	480	5	240	10	4800

**Table 2:** Experimental conditions for continuous mode.

No.	Power [W]	Irradiation Time [s]	Energy [J]
2-1	30	160	4800
2-2	60	80	4800
2-3	90	60	4800
2-4	120	40	4800
2-5	160	30	4800
2-6	240	20	4800

**Table 3:** Experimental conditions for different levels of power in the first stage of two-stage irradiation.

No.	Power of First Irradiation [W]	Irradiation Time of First Irradiation [s]	Power of Second Irradiation [W]	Irradiation Time of Second Irradiation [s]	Energy [J]
3-1 (1-2)	480	5	60	40	4800
3-2	320	7.5	60	40	4800
3-3	240	10	60	40	4800
3-4	120	20	60	40	4800

## Data Availability

No new data were created or analyzed in this study.
